# *Lacticaseibacillus paracasei* LP36 Alleviates Lipopolysaccharide-Induced Depression-like Behavior in Mice: A Serum Metabolomics Study

**DOI:** 10.3390/nu18152468

**Published:** 2026-07-29

**Authors:** Qiuyue Chen, Zhiyu Guo, Deyu Zhu, Fei Wang, Chenbi Sun, Rongrong Li, Dan Yin, Chunlu Li, Baijuan Xia, Weifen Li, Yixin Li

**Affiliations:** 1 Department of Histology and Embryology, School of Basic Medical Sciences, Guizhou Medical University, Gui’an New District, Guiyang 561113, China; chenqiuyuep@163.com (Q.C.); 15581211303@163.com (D.Z.); sunchenbi2026@163.com (C.S.); lee_rongrong@hotmail.com (R.L.); 13984018207@163.com (D.Y.); xiabaijuan4191@163.com (B.X.); liyixin@gmc.edu.cn (Y.L.); 2 School of Humanities, Guizhou Medical University, Gui’an New District, Guiyang 561113, China; juicefiish@163.com; 3 State Key Laboratory for Quality and Safety of Agro-Products, Institute of Agro-Product Safety and Nutrition, Zhejiang Academy of Agricultural Sciences, Hangzhou 310021, China; 22017007@zju.edu.cn; 4 Key Laboratory for Research on Autoimmune Diseases of Higher Education Schools in Guizhou Province, Guiyang 561113, China; 5 Institute of Animal Nutrition and Feed Sciences, College of Animal Sciences, Zhejiang University, Hangzhou 310058, China

**Keywords:** *Lacticaseibacillus paracasei* LP36, lipopolysaccharide, depression-like behavior, untargeted metabolomics, polyunsaturated fatty acids, gut microbiota

## Abstract

Background/Objectives: Major depressive disorder (MDD) is a highly prevalent and disabling psychiatric condition for which current therapies remain inadequate. Probiotics acting on the microbiota–gut–brain axis are a promising preventive strategy, but their benefits are strain-specific. Here we examined whether *Lacticaseibacillus paracasei* LP36 alleviates lipopolysaccharide (LPS)-induced depression-like behavior in mice, and we characterized its metabolic actions using serum untargeted metabolomics. Methods: Sixty male C57BL/6J mice were randomized into eight groups (58 completed the study and were analyzed) and received saline or LP36 (1 × 10^8^, 1 × 10^9^, or 1 × 10^10^ colony-forming units (CFU)/mL) by gavage for 28 days, with LPS (0.5 mg/kg, i.p.) or saline given during the last 10 days. Results: LP36 pretreatment alleviated LPS-induced depression-like behavior in the sucrose preference, forced swimming, and tail suspension tests. Of 2867 annotated serum metabolites, 404 differed between the LPS and control groups, and 601 between the LP36 (10^10^)-treated and LPS groups. Among 180 shared differential metabolites, 172 (95.6%) changed in opposite directions, indicating that LP36 mainly reverses LPS-induced metabolic perturbations. KEGG enrichment localized these effects to lipid-related pathways, with lower relative signal intensities of polyunsaturated fatty acids and sphingolipids under LPS and higher relative signal intensities after LP36. In addition, the relative signal intensities of several microbiota-derived metabolites, including indoles, kynurenines, and secondary bile acids, shifted toward control-group values after LP36 intervention, which might reflect a contribution of the gut microbiota. Conclusions: LP36 is thus a candidate psychobiotic that may alleviate depression by reshaping gut microbiota-related metabolism and the associated lipid remodeling.

## 1. Introduction

Major depressive disorder (MDD) is one of the most prevalent and disabling psychiatric disorders. It is characterized by persistent low mood, anhedonia, and cognitive and somatic impairment, and imposes a heavy burden on both patients and society [1]. The 2020 COVID-19 pandemic further aggravated this problem, with a marked global increase in MDD cases [2]. Antidepressant drugs and psychotherapy remain the clinical first-line options, but they are effective in only a subset of patients and are often accompanied by delayed onset of action, adverse effects, and a high risk of relapse [3,4]. Complementary strategies that can help prevent depression, or improve outcomes in the substantial proportion of patients who respond inadequately to current treatments, therefore remain an important unmet clinical need.

Among the mechanisms proposed for MDD, neuroinflammation has received increasing attention [3,5]. Patients with depression show elevated levels of pro-inflammatory cytokines in peripheral blood, and increases in tumor necrosis factor-α (TNF-α) and interleukin-6 (IL-6) have been confirmed by meta-analysis [6]; these inflammatory mediators can reach the brain and worsen depressive symptoms by affecting neurotransmitter metabolism, neuroplasticity, and blood–brain barrier permeability [7]. Lipopolysaccharide (LPS), the major component of the outer membrane of Gram-negative bacteria and a potent activator of the innate immune system, is widely used to construct inflammation-related animal models of depression; systemic administration of LPS reliably induces depression-like behaviors in mice, including reduced sucrose preference and prolonged immobility in the forced swimming and tail suspension tests [7,8]. The LPS-induced model thus provides a mechanistically defined and reproducible platform for examining anti-inflammatory and antidepressant interventions.

In parallel, the microbiota–gut–brain axis has become a focus of depression research as an important route linking the gut ecosystem to mood regulation [9,10,11]. Probiotics with mental-health benefits, sometimes termed “psychobiotics,” can favorably influence mood by restoring the balance of the gut microbiota and exerting anti-inflammatory effects, partly through microbial metabolites such as short-chain fatty acids [12,13]; meta-analyses of randomized controlled trials indicate that probiotic supplementation significantly lowers depression scale scores [14,15]. In various animal models of depression, probiotics have been shown to alleviate depression-like behavior and improve related biochemical and metabolic disturbances [8,16,17,18,19]. This evidence indicates that probiotic intervention targeting the gut microbiota is a promising strategy for the prevention and treatment of depression.

Among probiotics, *Lacticaseibacillus paracasei* (formerly *Lactobacillus paracasei*) is a widely used species, and several of its strains have shown antidepressant or neuroprotective activity in preclinical studies. *L. paracasei* PS23 reversed corticosterone-induced depression- and anxiety-like behaviors and restored hippocampal neurotrophic factor and monoamine neurotransmitter levels [20]; *L. paracasei* CCFM1229 alleviated chronic stress-induced depression-like behavior [21]; and heat-inactivated *L. paracasei* N1115 reduced the brain-function impairment caused by long-term antibiotic exposure [22]. It should be emphasized, however, that the health effects of probiotics are highly strain-specific, and the efficacy shown by one strain cannot be reliably extrapolated to other strains of the same species [23]; the antidepressant potential of any given *L. paracasei* strain therefore needs to be examined for that strain individually. Moreover, most existing studies of *L. paracasei* strains remain at the behavioral level, and the metabolic mechanisms underlying their antidepressant effects are still poorly understood.

*Lacticaseibacillus paracasei* LP36 is a strain originally isolated from “Suanshui,” a traditional Chinese fermented vegetable food, and selected for its favorable probiotic properties [19]. Its safety, together with good gastrointestinal tolerance, adhesion capacity, and antioxidant activity, has been confirmed by whole-genome sequencing and metabolomic analysis [24]. In liver-injury models, LP36 has been shown to modulate the gut microbiota and to counteract LPS-driven inflammation [19,25], precisely the actions through which probiotics are thought to influence mood via the microbiota–gut–brain axis. Its effect on depression-like behavior has not been tested, and the metabolic mechanisms involved remain unknown. Untargeted metabolomics can systematically describe metabolite changes after perturbation and has become a powerful tool for revealing the mechanisms of depression and its interventions [16,26]. Serum was selected as the analytical matrix because the primary objective was to characterize the integrated systemic metabolic response to LPS and LP36 and to interpret circulating metabolic changes alongside behavioral outcomes in the same experimental model. This choice was therefore directly aligned with the systemic and behavior-linked objective of the present study. On this basis, the present study combined an LPS-induced mouse model of depression with serum untargeted metabolomics, aiming to: (i) examine whether LP36 pretreatment protects mice from LPS-induced depression-like behavior; (ii) characterize the serum metabolic disturbances induced by LPS; and (iii) identify the key metabolic pathways and metabolites modulated by LP36. To our knowledge, this is the first study to systematically examine the antidepressant effect of *L. paracasei* LP36 and to characterize it at the metabolic level; the results provide a mechanistic basis for this strain as a gut-microbiota-targeted strategy for the prevention and treatment of depression.

## 2. Materials and Methods

### 2.1. Animals

Male C57BL/6J mice (8 weeks old) were purchased from the Animal Management Center of Guizhou Medical University (Guiyang, China). After one week of acclimatization, they were used for experiments at 9 weeks of age and a body weight of 20–25 g. Mice were housed under a 12 h light/dark cycle at 22–24 °C and 40–60% relative humidity, with free access to standard chow and water. All procedures were approved by the Animal Ethics Committee of Guizhou Medical University (approval No. 2400038) and were performed in accordance with the institutional guidelines for the care and use of laboratory animals.

### 2.2. Bacterial Strain, LPS, and Reagent Preparation

*Lacticaseibacillus paracasei* (formerly *Lactobacillus paracasei*) LP36 (strain accession No. CCTCC NO M20232287) was kindly provided as a freeze-dried powder by the laboratory of Prof. Weifen Li (Institute of Animal Nutrition and Feed Science, College of Animal Sciences, Zhejiang University). LP36 was originally isolated from “Suanshui,” a traditional Chinese fermented vegetable food, and its probiotic properties, whole-genome sequence, and safety have been characterized in previous studies [24,25]. Before administration, the LP36 freeze-dried powder was resuspended in sterile saline and adjusted to working concentrations of 1 × 10^8^, 1 × 10^9^, and 1 × 10^10^ CFU/mL. LPS (from *Escherichia coli*; catalog No. L2880) was purchased from Sigma-Aldrich (St. Louis, MO, USA) and freshly prepared in sterile saline before use.

### 2.3. Experimental Design and Grouping

Sixty male C57BL/6J mice were randomly assigned by body weight into eight groups, following a 2 × 4 factorial design with the first factor being intraperitoneal LPS injection (NS or LPS) and the second factor being the LP36 gavage dose (NS, 1 × 10^8^, 1 × 10^9^, or 1 × 10^10^ CFU/mL). The eight groups were NS + NS, NS + LPS, LP36 10^8^ + NS, LP36 10^9^ + NS, LP36 10^10^ + NS, LPS + LP36 10^8^, LPS + LP36 10^9^, and LPS + LP36 10^10^. Two mice assigned to the NS + LPS group died, one during the acclimatization period and one during the experimental period, leaving 58 mice for the final analysis: 5 in the NS + NS group, 5 in the NS + LPS group, and 8 in each of the remaining six groups. Group allocation used simple randomization with balancing for baseline body weight, and all animals came from a single batch, thereby limiting potential confounders. No a priori power calculation was performed; the group sizes were based on previous comparable LPS-induced depression studies and the 3Rs principle of animal use.

Each mouse received a daily oral gavage (0.2 mL) of the corresponding concentration of LP36 suspension or saline for 28 consecutive days. During the last 10 days of the gavage period, LPS (0.5 mg/kg) or an equal volume of sterile saline was injected intraperitoneally once each morning to establish the depression model. The sucrose preference test (SPT), forced swimming test (FST), and tail suspension test (TST) were assessed at three time points: at baseline before gavage on Day 0, before LPS injection on Day 18, and after LPS treatment on Day 28.

### 2.4. Behavioral Tests

Before each test, mice were acclimatized to the testing room for at least 1 h, and all tests were performed under standardized lighting and low-noise conditions. Behavioral activity was recorded with a video tracking system (Tracking Master, Beijing Zhongshi Dichuang Technology Co., Ltd., Beijing, China). The SPT, FST, and TST were assessed in parallel at the three time points. Group allocation was known to the personnel who performed the gavage and LPS injections, as required for dosing; however, during behavioral testing and outcome assessment the assessors were blinded to group allocation. Immobility in the FST and TST was scored automatically by the video-tracking system, and sucrose preference was derived from measured fluid intake, further limiting subjective bias. The primary outcome measures were the behavioral indices—sucrose preference in the SPT and immobility time in the FST and TST—whereas serum untargeted metabolomics served as the secondary, molecular outcome.

#### 2.4.1. Sucrose Preference Test (SPT)

The SPT was used to assess anhedonia, following the protocol originally established by Willner et al. [27] with minor modifications. Mice were first habituated to two bottles of 1–2% sucrose solution for 24 h, then given one bottle of 1–2% sucrose solution and one bottle of water for 24 h, with bottle positions switched after 12 h. After 24 h of water deprivation, each mouse was given one bottle of 1–2% sucrose solution and one bottle of water; bottle positions were switched after 12 h, and intake over 24 h was recorded. Sucrose preference was calculated as sucrose intake/(sucrose intake + water intake).

#### 2.4.2. Forced Swimming Test (FST)

The FST was used to assess depression-like immobility, following the protocol originally described by Porsolt et al. [28]. Each mouse was placed for 6 min in a transparent cylindrical container (10 cm diameter, 20 cm height) filled with water (23 ± 1 °C, 13 cm depth), and immobility time during the last 4 min was recorded.

#### 2.4.3. Tail Suspension Test (TST)

The TST was performed following the protocol originally described by Steru et al. [29]. Each mouse was suspended individually upside down by tape (applied approximately 1 cm from the tail tip) for 6 min, and immobility time during the last 4 min was recorded. Mice that climbed their own tails were excluded from the analysis.

### 2.5. Serum Sample Collection

After the final behavioral test on Day 28, 3 mice were randomly selected from each group (24 in total, *n* = 3 per group) for serum untargeted metabolomics. Three biological replicates per group is a common design for exploratory untargeted serum metabolomics and was adopted here to balance analytical cost against the discovery-oriented aim of the study; this small sample size is a recognized limitation of the metabolomic analysis. Mice were anesthetized with intraperitoneal tribromoethanol and blood was collected; after clotting, serum was obtained by centrifugation and stored at −80 °C until analysis.

### 2.6. Serum Untargeted Metabolomics

Serum untargeted metabolomics was performed by Novogene Co., Ltd. (Beijing, China). For metabolite extraction, each serum sample (100 µL) was resuspended in pre-cooled 80% methanol by vortexing, incubated on ice for 5 min, and centrifuged at 15,000× *g* and 4 °C for 20 min. The supernatant was diluted with LC-MS-grade water to a final methanol concentration of 53%, transferred to a fresh tube, and centrifuged again at 15,000× *g* and 4 °C for 20 min; the resulting supernatant was used for analysis. Quality control (QC) samples were prepared by pooling equal volumes of all study samples and were injected at the beginning, middle, and end of the analytical sequence to monitor system stability. The study samples were analyzed within a single analytical batch, and although their injection order was not separately recorded, the repeated QC injections monitored instrument stability across the analytical sequence, and the high QC reproducibility (Section 3.2) indicates that analytical drift and batch effects had a limited impact on the reported data.

Chromatographic separation was performed on a Vanquish ultra-high-performance liquid chromatography (UHPLC) system (Thermo Fisher Scientific, Germering, Germany) with a Hypersil Gold C18 column (100 × 2.1 mm, 1.9 µm; Thermo Fisher Scientific, Waltham, MA, USA), running a 12 min linear gradient at a flow rate of 0.2 mL/min. Mobile phase A was 0.1% formic acid in water and mobile phase B was methanol; the gradient was 2% B held for 1.5 min, increased to 85% B at 3 min, increased to 100% B at 10 min, returned to 2% B at 10.1 min, and held at 2% B until 12 min. Mass spectrometric detection was performed on an Orbitrap Exploris 120 mass spectrometer (Thermo Fisher Scientific, Bremen, Germany) in both positive and negative electrospray ionization modes, with a spray voltage of 3.5 kV, capillary temperature of 320 °C, sheath gas flow rate of 35 psi, auxiliary gas flow rate of 10 L/min, S-lens RF level of 60, and auxiliary gas heater temperature of 350 °C.

Raw data were processed with XCMS for peak alignment, extraction, and quantification, with the mass deviation set to 10 ppm. Metabolites were identified by matching against Novogene’s in-house high-quality MS/MS database (NovoMetDB). Metabolites with more than 50% missing values were removed, and the remaining missing values were imputed using the K-nearest neighbors (KNN) algorithm; background ions were subtracted based on blank samples, the data were normalized to relative peak area, and compounds with a coefficient of variation greater than 30% in the QC samples were removed. Identified metabolites were annotated against the KEGG, HMDB, and LIPID MAPS databases. A total of 1512 and 1355 metabolites were identified in positive and negative ion modes, respectively, for a total of 2867. Because these annotations were based on MS/MS spectral-library matching (NovoMetDB) without co-analysis of authentic chemical standards, they correspond to level 2 (putatively annotated compounds) or below of the Metabolomics Standards Initiative rather than level-1 identifications [30]; accordingly, the reported values represent relative signal abundances (normalized to relative peak area) rather than absolute concentrations.

### 2.7. Data Processing and Statistical Analysis

Behavioral data are expressed as mean ± standard deviation (SD). The SPT, FST, and TST data at the three time points were compared using 2 × 4 two-way analysis of variance (ANOVA; factors: injection × dose), with Tukey–Kramer post hoc tests. Homogeneity of variance across the eight Day-28 groups was assessed with Levene’s test (median-centered) before ANOVA, and the assumption was met for all three outcomes (SPT *p* = 0.62, FST *p* = 0.87, TST *p* = 0.86; Table S2). Comparisons of the same mouse between time points used paired *t*-tests. All behavioral statistics were performed with IBM SPSS Statistics (v26) and GraphPad Prism (v8.0.2).

Metabolomic data were analyzed with Python 3.12 and the scikit-learn library (v1.4) for principal component analysis (PCA) and partial least squares-discriminant analysis (PLS-DA) to visualize metabolic differences between groups; intensity data were first log_2_(x + 1)-transformed, mean-centered, and unit-variance-scaled. Because PLS-DA is prone to overfitting and requires rigorous validation [31], the performance of the PLS-DA models was described by the cross-validated Q^2^ value, with Q^2^ > 0.5 commonly cited as a reference level in untargeted metabolomics [26]. The statistical significance (*p* value) of each metabolite was determined by an independent-samples *t*-test, and the fold change (FC) was calculated as the ratio of the mean quantitative values between the compared groups. Differential metabolites were screened using the combined thresholds of variable importance in projection (VIP) > 1.0, FC > 1.2 or FC < 0.833 (i.e., |log_2_FC| > 0.263), and *p* < 0.05. KEGG pathway enrichment analysis was used to interpret the biological relevance of the differential metabolites; pathways with an enrichment *p* value < 0.05 were considered significantly enriched. *p* < 0.05 was considered statistically significant.

## 3. Results

### 3.1. LP36 Alleviates LPS-Induced Depression-like Behavior

**Figure 1:**
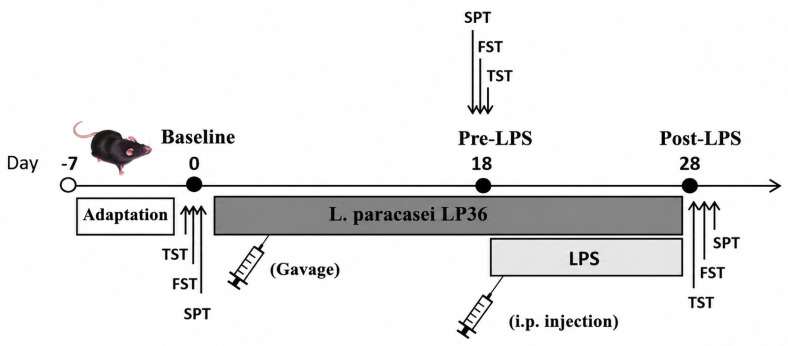
Experimental timeline. Days −7 to 0 were the acclimatization period. After behavioral baseline testing on Day 0, daily gavage of *Lacticaseibacillus paracasei* LP36 began and continued until Day 28. The second behavioral measurement was taken on Day 18, after which 10 consecutive days of intraperitoneal LPS injection began, and the third behavioral measurement was completed on Day 28. The SPT, FST, and TST were collected in parallel at the three time points.

A total of 58 mice were included in the Day 28 endpoint analysis. The healthy control group and the LPS depression-model group each comprised 5 mice, and the remaining six groups each comprised 8 mice. The eight groups followed a 2 × 4 factorial design, with the first factor being LPS injection and the second being the LP36 gavage dose at four levels (NS, 1 × 10^8^, 1 × 10^9^, and 1 × 10^10^ CFU). Behavioral data were collected at three time points: baseline on Day 0, before LPS injection on Day 18, and at the Day 28 endpoint. The three indicators were sucrose preference (SPT), forced swimming immobility time (FST), and tail suspension immobility time (TST). Day 28 endpoint data were analyzed by two-way ANOVA with Tukey–Kramer post hoc tests. The overall experimental schedule is shown in Figure 1.

**Figure 2:**
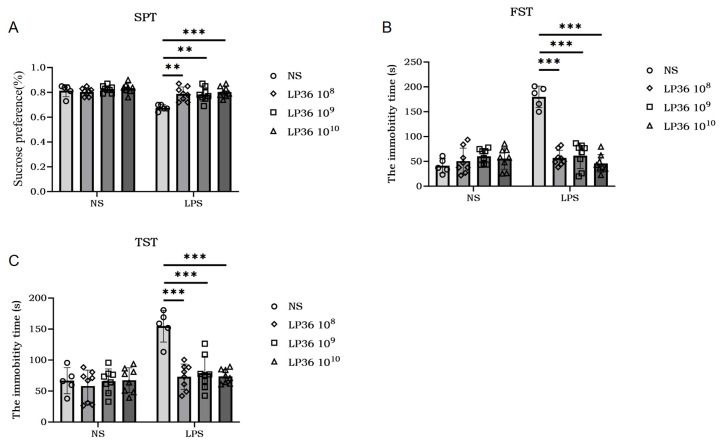
Effect of LP36 pretreatment on LPS-induced depression-like behavior. Sucrose preference (**A**), forced swimming immobility time (**B**), and tail suspension immobility time (**C**) of the eight groups at the Day 28 endpoint. In each panel, the left (NS) column shows the four gavage groups without LPS injection, from left to right NS, LP36 1 × 10^8^, 1 × 10^9^, and 1 × 10^10^ + NS; the right (LPS) column shows the four corresponding gavage groups with LPS injection. Bars show the mean, error bars show SD, and dots show individual values. Statistics used 2 × 4 two-way ANOVA with Tukey–Kramer post hoc tests. Significance markers indicate comparisons of the three LP36 dose groups versus the NS + LPS group within the LPS column; ** *p* < 0.01, *** *p* < 0.001.

We first confirmed whether the LPS depression-like behavior model was successfully established. As shown in Figure 2A, sucrose preference in the LPS model group decreased from 0.81 in the healthy control group to 0.68. Figure 2B shows that forced swimming immobility time in the LPS model group increased from 41.1 s to 180.0 s, and in Figure 2C the tail suspension immobility time also increased from 66.7 s to 154.7 s. The injection main effect in the two-way ANOVA was significant for all three indicators, with the corresponding F and *p* values listed in Table S2, indicating that the LPS depression model was successfully established.

We next examined whether LP36 gavage alone had any adverse effect on healthy mice. As shown in the NS column of Figure 2, sucrose preference for the three LP36-alone groups and the control group ranged from 0.80 to 0.83, forced swimming immobility time from 51 to 60 s, and tail suspension immobility time from 58 to 68 s. The between-group differences for all three indicators fell within the range of within-group variation and showed no dose-dependent shift, indicating that the dose range of 1 × 10^8^–1 × 10^10^ CFU had acceptable safety in healthy mice; the complete paired *t*-test results are provided in Table S1.

Finally, we assessed whether LP36 pretreatment alleviated LPS-induced depression-like behavior. As shown in the LPS column of Figure 2A, the three doses of LP36 + LPS raised sucrose preference from 0.68 in the NS + LPS group to 0.79, 0.78, and 0.80, with all three comparisons reaching significance in Tukey–Kramer post hoc tests. Figure 2B shows that the three doses significantly reduced forced swimming immobility time from 180.0 s to 56.9, 61.4, and 45.9 s, and in Figure 2C the tail suspension immobility time also decreased significantly from 154.7 s to 73.0, 78.9, and 74.0 s. The interaction term in the two-way ANOVA was significant for all three indicators, with the complete F, *p*, and Tukey–Kramer post hoc comparisons provided in Table S2, indicating that the behavioral benefit of LP36 depended on whether the LPS challenge occurred. No statistical differences were observed among the three doses; the 1 × 10^10^ CFU dose was numerically closest to the control baseline for all three indicators, suggesting a trend toward better alleviation with high-dose pretreatment.

### 3.2. Quality Control and Overall Profile of the Serum Metabolome

**Figure 3:**
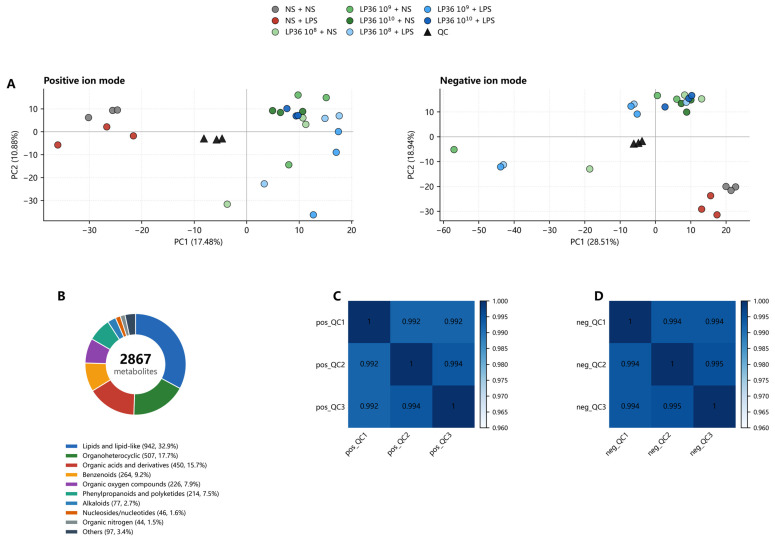
Quality control and overall profile of the serum metabolome. (**A**) PCA overview of the 24 serum samples and 3 QC samples in positive and negative ion modes; (**B**) first-level category composition of the metabolites after merging positive and negative ion modes; (**C**,**D**) Pearson correlation heatmaps of the 3 QC samples in positive and negative ion modes. POS, positive ion mode; NEG, negative ion mode.

Untargeted LC-MS was used to profile 24 serum samples (*n* = 3 per group), with data acquired in both positive and negative ion modes. A total of 1512 metabolites were annotated in positive ion mode and 1355 in negative ion mode, for a total of 2867; the sample intensity matrix for all metabolites is provided in Table S3. By classification, lipids and lipid-like molecules were the most abundant, with 942 (32.9%), followed by organoheterocyclic compounds with 507 (17.7%) and organic acids and derivatives with 450 (15.7%). As shown in Figure 3B, lipid-related metabolites accounted for a relatively high proportion of the serum metabolites in this batch.

Within-batch reproducibility was assessed using pooled QC samples. Figure 3A shows that the three QC samples clustered closely in the PCA plots for both positive and negative ion modes; Figure 3C,D show that the Pearson correlation coefficients among the QC samples were 0.992–0.994 and 0.994–0.995, respectively, all above 0.95. These results indicate that the LC-MS analysis in this batch was stable and the data reproducibility was good.

Subsequent analyses focused on two core comparisons: first, the NS + LPS group versus the NS + NS group, to determine the serum metabolic changes induced by LPS treatment; and second, the LPS + LP36 10^10^ group versus the NS + LPS group, to observe whether the related metabolic changes after high-dose LP36 intervention were closer to the normal state. Although the differential analysis focused on the highest dose (1 × 10^10^ CFU), which showed the strongest trend toward behavioral normalization (Section 3.1), the serum metabolic response was consistent across doses: the number of differential metabolites increased with dose (494, 533, and 601 for the 1 × 10^8^, 1 × 10^9^, and 1 × 10^10^ groups versus NS + LPS), the fold-changes of the two lower-dose comparisons were strongly correlated with those at 1 × 10^10^ (Spearman r = 0.95 and 0.92, respectively), and the key lipid mediators were concordantly up-regulated at all three doses (Figure S1). The 1 × 10^10^ dose was therefore analyzed as a representative dose rather than a dose-specific effect.

### 3.3. The Serum Metabolome Changes After LPS Induction

To further examine the effect of LPS treatment on peripheral metabolism, a differential analysis of the serum untargeted metabolome was performed between the NS + LPS group and the NS + NS group.

**Figure 4:**
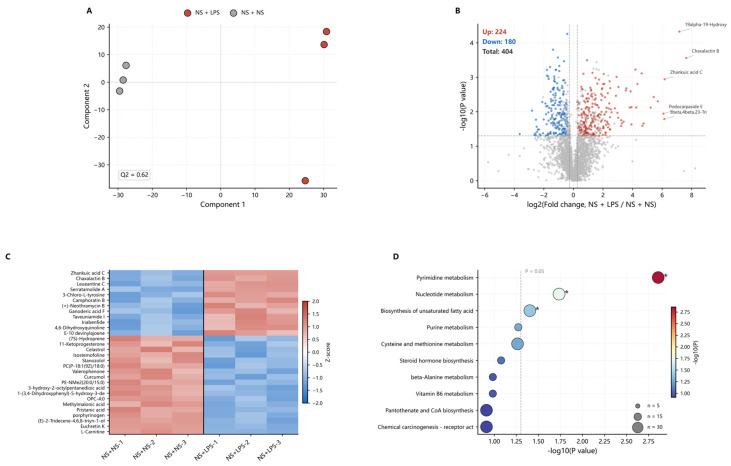
Serum differential metabolite analysis between the NS + LPS group and the control group. (**A**) PLS-DA score plot based on merged POS and NEG features; (**B**) volcano plot of differential metabolites; (**C**) heatmap of the top 30 differential metabolites; (**D**) KEGG enrichment bubble plot. PLS-DA used *n* = 3 per group. Differential screening criteria were VIP > 1, FC > 1.2 or FC < 0.833, and *p* < 0.05. In (**B**), the dashed lines indicate the screening thresholds (|log2FC| = 0.263; *p* = 0.05), and two metabolite names are abbreviated for clarity: “19alpha-19-Hydroxy” denotes 19alpha-19-Hydroxy-3,11-dioxo-12-ursen-28-oic acid, and “3beta,4beta,23-Tri” denotes 3beta,4beta,23-Trihydroxy-24,30-dinor-olean-12,20(29)-dien-28-oic acid; the full list of differential metabolites is provided in Table S4. In (**D**), asterisks denote pathways with an enrichment *p* < 0.05.

As shown in Figure 4A, the serum samples of the NS + LPS and NS + NS groups (*n* = 3 per group) were distributed in different regions of the PLS-DA score plot; the model *Q*^2^ was 0.62, above the *Q*^2^ > 0.5 reference level commonly used in untargeted metabolomics [26].

Throughout the Results, all reported metabolite differences, including up-/down-regulation and fold-change values, refer to normalized relative signal intensities rather than absolute concentrations. Using VIP > 1, FC > 1.2 or FC < 0.833, and *p* < 0.05 as screening criteria, 404 differential metabolites were identified between the NS + LPS and NS + NS groups, of which 224 were upregulated and 180 downregulated. Figure 4B shows the overall distribution of these differential metabolites, and the complete list is provided in Table S4.

As shown in Figure 4C, the top 30 differential metabolites showed distinct abundance patterns between the NS + LPS and NS + NS groups. By category, lipids and lipid-like molecules contributed the most, with 137 (58 upregulated and 79 downregulated), indicating that this category changed substantially after LPS treatment and was predominantly downregulated; organic acids and derivatives and organoheterocyclic compounds also accounted for a high proportion (Table S7).

KEGG enrichment analysis identified three significant pathways among the top 10: pyrimidine metabolism (*p* = 0.001), nucleotide metabolism (*p* = 0.018), and biosynthesis of unsaturated fatty acids (*p* = 0.039); purine metabolism and cysteine and methionine metabolism were in the borderline-significant range (*p* = 0.053 and 0.054). These results are shown in Figure 4D and Table S5. The findings indicate that the LPS stage involved changes in pathways such as nucleotide metabolism and biosynthesis of unsaturated fatty acids; lipid-related pathways had already appeared at this stage but were not yet the most concentrated direction of enrichment, an issue examined further in the section on LPS + LP36 10^10^ intervention.

### 3.4. The Serum Metabolome Is Reshaped After LPS + LP36 10^10^ Intervention

To evaluate the regulatory effect of high-dose LP36 on the serum metabolome under LPS challenge, a differential analysis was performed between the LPS + LP36 10^10^ group and the NS + LPS group.

**Figure 5:**
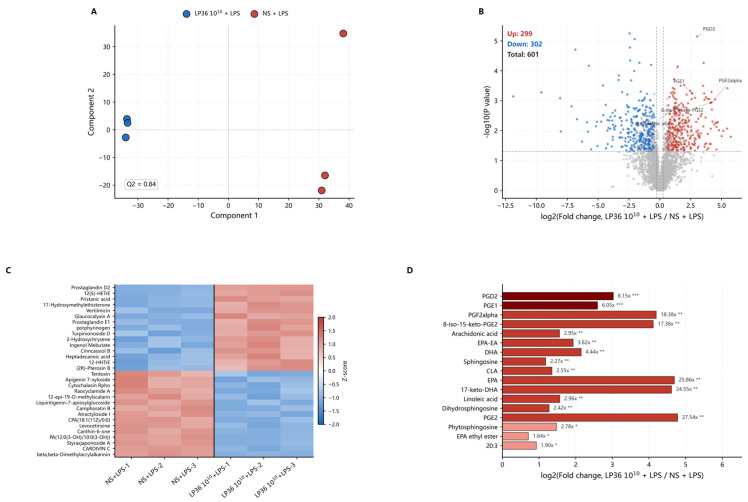
Serum differential metabolite analysis between the LP36 10^10^ + LPS group and the NS + LPS group. (**A**) PLS-DA score plot based on merged POS and NEG features; (**B**) volcano plot of differential metabolites; (**C**) heatmap of the top 30 differential metabolites; (**D**) log2FC of key lipid metabolites; * *p* < 0.05, ** *p* < 0.01, *** *p* < 0.001, and the bar colours correspond to these discrete significance categories: light red, 0.01 ≤ *p* < 0.05; medium red, 0.001 ≤ *p* < 0.01; dark red, *p* < 0.001. PLS-DA used *n* = 3 per group, and Q2 was calculated by cross-validation. In (**B**), the dashed lines indicate the screening thresholds (|log2FC| = 0.263; *p* = 0.05). Differential screening criteria were VIP > 1, FC > 1.2 or FC < 0.833, and *p* < 0.05.

As shown in Figure 5A, the serum samples of the LPS + LP36 10^10^ and NS + LPS groups were distributed in different regions of the PLS-DA score plot, with a model *Q*^2^ of 0.84, above the *Q*^2^ > 0.5 reference level.

Using the same screening criteria as in Section 3.3 (VIP > 1, FC > 1.2 or FC < 0.833, *p* < 0.05), 601 differential metabolites were identified between the LPS + LP36 10^10^ and NS + LPS groups, of which 299 were upregulated and 302 downregulated. This number exceeds the 404 differential metabolites attributable to LPS perturbation in Section 3.3, and the numbers of up- and downregulated metabolites were similar, indicating that the serum metabolome underwent an overall reshaping rather than a unidirectional recovery after high-dose LP36 intervention. These results are shown in Figure 5B and Table S6.

As shown in Figure 5C, the top 30 differential metabolites showed distinct abundance patterns between the LPS + LP36 10^10^ and NS + LPS groups. By category, lipids and lipid-like molecules contributed the most, with 245 (141 upregulated and 104 downregulated), predominantly upregulated; this contrasts with the predominance of lipid downregulation at the LPS stage in Section 3.3. Organoheterocyclic compounds totaled 103 (40 upregulated and 63 downregulated) (Table S7).

Focusing on the key lipid metabolites shown in Figure 5D, prostaglandin D2 was upregulated 8.15-fold (*p* = 7.1 × 10^−6^), prostaglandin E1 6.05-fold (*p* = 5.6 × 10^−4^), prostaglandin F2α 18.38-fold (*p* = 1.1 × 10^−3^), and prostaglandin E2 27.54-fold (*p* = 9.7 × 10^−3^). Polyunsaturated fatty acid-related metabolites were also upregulated, including EPA 25.86-fold (*p* = 5.3 × 10^−3^), 17-keto-DHA 24.55-fold (*p* = 5.4 × 10^−3^), DHA 4.44-fold (*p* = 1.7 × 10^−3^), eicosapentaenoyl ethanolamide 3.82-fold (*p* = 1.6 × 10^−3^), arachidonic acid 2.95-fold (*p* = 1.4 × 10^−3^), linoleic acid 2.96-fold (*p* = 5.7 × 10^−3^), and conjugated linoleic acid 2.55-fold (*p* = 2.6 × 10^−3^). Among sphingolipid-related metabolites, sphingosine, dihydrosphingosine, and phytosphingosine were upregulated 2.27-fold (*p* = 2.1 × 10^−3^), 2.42-fold (*p* = 9.5 × 10^−3^), and 2.78-fold (*p* = 1.0 × 10^−2^), respectively. These results indicate that the coordinated upregulation of polyunsaturated fatty acids, prostaglandins, and sphingolipids is a representative feature of serum metabolic reshaping under high-dose LP36 intervention; the corresponding pathway analysis is presented in Section 3.5.

### 3.5. KEGG Pathway Enrichment of LP36 Intervention Is Dominated by Lipid-Related Pathways

To analyze the pathway-level changes of high-dose LP36 intervention, KEGG enrichment analysis was performed on the 601 differential metabolites from Section 3.4.

**Figure 6:**
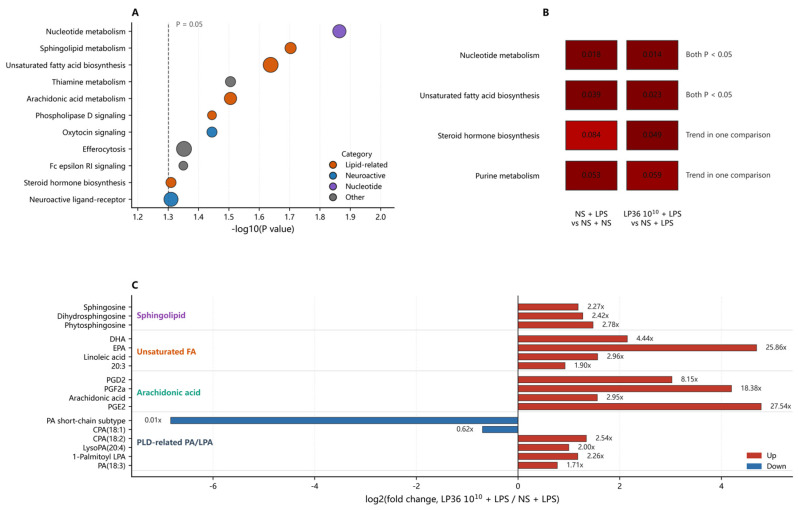
KEGG pathway enrichment after high-dose LP36 intervention and changes in lipid-related metabolites. (**A**) KEGG enrichment bubble plot of the differential metabolites between the LPS + LP36 10^10^ group and the NS + LPS group; bubble size indicates the number of hit metabolites and color indicates the pathway category. (**B**) Pathways that were commonly significant or borderline-significant in the NS + LPS vs. NS + NS and LP36 10^10^ + LPS vs. NS + LPS comparisons; nucleotide metabolism and biosynthesis of unsaturated fatty acids reached *p* < 0.05 in both comparisons; the enrichment *p* value is printed in each cell, and the colour intensity reflects its magnitude (darker red indicates a smaller p value). (**C**) Representative metabolite changes in four classes of lipid-related pathways or related lipid species; red indicates upregulation and blue indicates downregulation.

As shown in Figure 6A, 11 significantly enriched pathways were obtained at the *p* < 0.05 threshold (Table S8). Among them, five lipid-related pathways constituted the main direction of enrichment, including sphingolipid metabolism (*p* = 0.020), biosynthesis of unsaturated fatty acids (*p* = 0.023), arachidonic acid metabolism (*p* = 0.031), the phospholipase D signaling pathway (*p* = 0.036), and steroid hormone biosynthesis (*p* = 0.049). Two neuroactivity-related pathways were enriched, the oxytocin signaling pathway (*p* = 0.036) and neuroactive ligand-receptor interaction (*p* = 0.049). The remaining four covered nucleotide metabolism (*p* = 0.014), thiamine metabolism (*p* = 0.031), efferocytosis (*p* = 0.044), and the Fc epsilon RI signaling pathway (*p* = 0.045), spanning the nucleic-acid, vitamin, and immune dimensions.

The KEGG enrichment from the NS + LPS versus NS + NS comparison in Section 3.3 was then intersected with the KEGG enrichment of LP36 intervention in this section. As shown in Figure 6B, four pathways were simultaneously significantly or borderline-significantly enriched in both comparisons: nucleotide metabolism (LPS *p* = 0.018; LP36 *p* = 0.014), biosynthesis of unsaturated fatty acids (LPS *p* = 0.039; LP36 *p* = 0.023), steroid hormone biosynthesis (LPS *p* = 0.084; LP36 *p* = 0.049), and purine metabolism (LPS *p* = 0.053; LP36 *p* = 0.059). Biosynthesis of unsaturated fatty acids was the only lipid pathway that reached the *p* < 0.05 threshold in both comparisons; together with the overall pattern of lipid downregulation in Section 3.3 (58 upregulated, 79 downregulated) and lipid upregulation in Section 3.4 (141 upregulated, 104 downregulated), this indicates a directional regulation of this pathway under high-dose LP36 intervention.

Focusing further on the representative metabolite composition of the four core lipid-related pathways, Figure 6C shows that, in the sphingolipid metabolism pathway, sphingosine, dihydrosphingosine, and phytosphingosine were all upregulated; in the biosynthesis of unsaturated fatty acids pathway, EPA, DHA, linoleic acid, and eicosatrienoic acid were all upregulated; in the arachidonic acid metabolism pathway, arachidonic acid was upregulated together with prostaglandin D2, prostaglandin E2, and prostaglandin F2α; and among the phospholipase D-related PA/LPA metabolites, there were both upregulated metabolites, such as LysoPA, CPA(18:2), and 1-palmitoyl LPA, and significantly downregulated short-chain PA subtypes and CPA(18:1), showing a mixed direction.

These results indicate that high-dose LP36 intervention was dominated by enrichment of lipid-related pathways at the level of the serum metabolome, with biosynthesis of unsaturated fatty acids and arachidonic acid metabolism affected by both LPS perturbation and LP36 intervention; whether individual metabolites showed opposite-direction changes in the two comparisons required further analysis.

### 3.6. LP36 and LPS Show Opposite-Direction Changes in Shared Differential Metabolites

To evaluate whether the metabolic effects of high-dose LP36 intervention corresponded in direction to the metabolic changes induced by LPS, the 404 differential metabolites from the NS + LPS versus NS + NS comparison in Section 3.3 were intersected with the 601 differential metabolites from the LPS + LP36 10^10^ versus NS + LPS comparison in Section 3.4.

**Figure 7:**
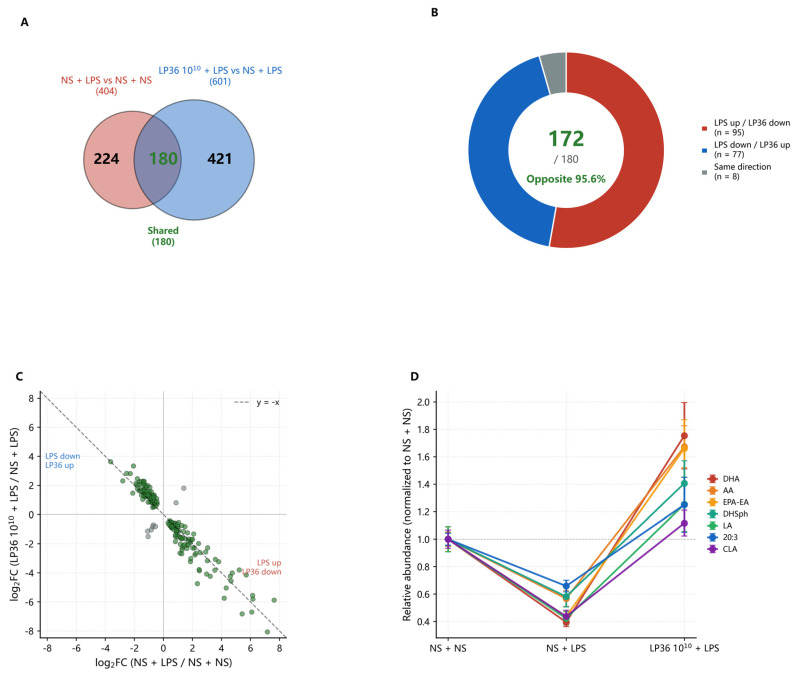
Metabolic closed loop in which LP36 and LPS show opposite-direction changes in shared differential metabolites. (**A**) Venn diagram of the differential metabolites from LPS vs. control (404) and LPS + LP36 10^10^ vs. LPS (601), with 180 common differential metabolites. (**B**) Doughnut chart classifying the 180 common differential metabolites by direction of change; 172 changed in opposite directions (95.6%), of which 95 showed higher relative signal intensity at the LPS stage followed by a downward shift after LP36 intervention and 77 showed lower relative signal intensity at the LPS stage followed by an upward shift after LP36 intervention; 8 changed in the same direction. (**C**) Anti-diagonal scatter plot of the log2FC of the 180 common metabolites, in which green circles denote opposite-direction metabolites (*n* = 172) and grey circles same-direction metabolites (*n* = 8); the dashed line is the y = −x reference. (**D**) Relative abundance trajectories of polyunsaturated fatty acid and sphingolipid representatives among the seven common metabolites (DHA, arachidonic acid, eicosapentaenoyl ethanolamide, linoleic acid, conjugated linoleic acid, dihydrosphingosine, and eicosatrienoic acid).

As shown in Figure 7A, the two comparisons shared 180 common differential metabolites, with 224 metabolites appearing only in the LPS perturbation and 421 only in the LP36 intervention.

The 180 common metabolites were classified by their direction of change in the two comparisons, as shown in Figure 7B. Of these, 172 (95.6%) changed in opposite directions and could be divided into two classes: 95 showed higher relative signal intensity at the LPS stage followed by a downward shift after LP36 intervention, and 77 showed lower relative signal intensity at the LPS stage followed by an upward shift after LP36 intervention; the remaining 8 metabolites changed in the same direction in the two comparisons.

Plotting the log_2_FC (NS + LPS/NS + NS) and log_2_FC (LP36 10^10^ + LPS/NS + LPS) of the 180 common metabolites in two-dimensional space, Figure 7C shows that the point set was distributed approximately along the y = −x anti-diagonal, further reflecting the correspondence of the metabolite change directions in the two comparisons; the few same-direction metabolites were located in the first and third quadrants.

To more directly show the relative abundance changes of individual metabolites across the three states of NS + NS, NS + LPS, and LP36 10^10^ + LPS, seven representative metabolites belonging to the 180 common metabolites and involving polyunsaturated fatty acids or sphingolipids were selected for a trajectory plot (Figure 7D). As shown in Figure 7D, the relative signal intensities of DHA, arachidonic acid, eicosapentaenoyl ethanolamide, linoleic acid, conjugated linoleic acid, dihydrosphingosine, and eicosatrienoic acid were 40–66% of the NS + NS group mean in the NS + LPS group and 112–175% of the NS + NS group mean in the LPS + LP36 10^10^ group, showing a V-shaped trajectory (an initial downward shift followed by an upward shift), with several metabolites exceeding the NS + NS group mean after LP36 intervention.

These results indicate that, among the identified differential metabolites, most common metabolites changed in opposite directions between LPS perturbation and LP36 intervention; the shift in relative signal intensity of polyunsaturated fatty acid- and sphingolipid-related metabolites toward or above the NS + NS group mean after LP36 intervention provides a metabolic-level correspondence to the alleviation of LPS-induced depression-like behavior by LP36 in Section 3.1. The complete direction-annotation table is provided in Table S9.

## 4. Discussion

This study showed at the behavioral level that LP36 pretreatment alleviated LPS-induced depression-like behavior in mice. On this basis, serum untargeted metabolomics showed that the metabolic effect of LP36 is mainly a reverse regulation of the LPS-induced perturbation, concentrated at the level of lipid metabolism (Section 3; Figure 5), consistent with previous meta-analyses identifying lipid metabolic disturbance as an important metabolic feature of depression [32].

Among the reversed metabolites, the changes in polyunsaturated fatty acids (PUFAs) and sphingolipids were the most prominent. The relative signal intensities of DHA, arachidonic acid, eicosapentaenoyl ethanolamide, linoleic acid, conjugated linoleic acid, dihydrosphingosine, and eicosatrienoic acid were 40–66% of the NS + NS group mean in the NS + LPS group and 112–175% of the NS + NS group mean after LP36 intervention, with some exceeding the NS + NS group mean. PUFAs are key components of neuronal membranes and participate in neurotransmission and inflammatory regulation through their derived mediators [33]. Clinical and animal studies indicate that low omega-3 PUFA levels are associated with depression risk and that EPA and DHA supplementation improves depressive symptoms [34], with their derived specialized pro-resolving mediators promoting the resolution of inflammation [35,36]. The PUFAs showing upward shifts in relative signal intensity with LP36 in this study covered both the omega-3 and omega-6 families, consistent with a return of lipid balance toward a more favorable direction [37]. He et al. and Mo et al. also reported disturbances in related lipid pathways in LPS depression models [8,16]. As an oral probiotic, LP36 may act indirectly, through gut microbiota-mediated regulation of lipid metabolism [38].

After LP36 intervention, several downstream products of the arachidonic acid pathway were markedly upregulated. Prostaglandin D2 was upregulated 8.15-fold (*p* = 7.1 × 10^−6^), prostaglandin E1 6.05-fold (*p* = 5.6 × 10^−4^), prostaglandin E2 27.54-fold (*p* = 9.7 × 10^−3^), prostaglandin F2α 18.38-fold (*p* = 1.1 × 10^−3^), and arachidonic acid itself 2.95-fold (*p* = 1.4 × 10^−3^). Prostaglandins have phase-dependent roles in the resolution of inflammation [39,40], and the marked upregulation of the prostaglandin profile observed here suggests that LP36 may promote the generation of resolution-phase lipid mediators; this remains correlative evidence and requires further validation by targeted lipidomics. Consistent with this interpretation, ω-3-derived specialized pro-resolving mediators such as resolvins produce antidepressant-like effects in rodent models of depression [41]. The coordinated upward shift in the relative signal intensities of polyunsaturated fatty acids and the marked up-regulation of prostaglandins under LP36 may therefore shift the lipid milieu toward a pro-resolving, inflammation-terminating state, linking the observed lipid remodeling to the anti-inflammatory action of the strain. In parallel, the concordant up-regulation of the sphingoid bases sphingosine, dihydrosphingosine, and phytosphingosine points to active remodeling of sphingolipid metabolism; as ceramide and related sphingolipids are elevated in the plasma of patients with depression [42,43], a shift of this pathway away from the pro-inflammatory ceramide axis would be consistent with the anti-inflammatory direction of the other lipid changes.

The KEGG enrichment results localized the metabolic regulation by LP36 to lipid-related pathways. Eleven pathways were significantly enriched in the LP36 versus LPS comparison, of which five lipid-related pathways formed the main part, including sphingolipid metabolism (*p* = 0.020), biosynthesis of unsaturated fatty acids (*p* = 0.023), arachidonic acid metabolism (*p* = 0.031), the phospholipase D signaling pathway (*p* = 0.036), and steroid hormone biosynthesis (*p* = 0.049). Biosynthesis of unsaturated fatty acids was the only lipid pathway that reached *p* < 0.05 in both the LPS-versus-control and LP36-versus-LPS comparisons, indicating a directional regulation under LP36 intervention. The sphingolipid metabolite ceramide is elevated in the plasma of patients with depression [42,43], and the phospholipase D pathway participates in the regulation of inflammatory responses [44,45], with inhibition of phospholipase D reducing neuroinflammation after central nervous system injury [46]. These metabolite alterations are consistent with the anti-inflammatory action of LP36 demonstrated in other disease models. LP36 lowered circulating pro-inflammatory factors in a mouse model of alcoholic liver disease [19] and suppressed the TLR4/NF-κB/MAPK pathway and the NLRP3 inflammasome in a D-GalN/LPS model of acute liver injury [25]. The NLRP3 inflammasome is a convergence node by which peripheral inflammation is converted into neuroinflammation, and NLRP3-driven pathways have been linked to the pathophysiology of depression [47]; the present metabolomic findings extend the anti-inflammatory capacity of LP36 to the level of lipid metabolism in an LPS-induced model of depression.

Beyond lipid-related pathways, neuroactive ligand-receptor interaction (*p* = 0.049) and the oxytocin signaling pathway (*p* = 0.036) were also significantly enriched after LP36 intervention. Disturbances of these pathways have been reported to be associated with depression- and anxiety-like behaviors [48], although the evidence at the neural-signaling level in this study remains relatively indirect.

Beyond the reverse regulation at the lipid level, the serum metabolic profile in this study further indicates that the antidepressant effect of LP36 is associated with the metabolic activity of the gut microbiota. As an orally administered probiotic, LP36 does not itself enter the circulation, and its influence on the serum metabolome is generally achieved by reshaping the gut microbiota and altering the microbial metabolites that enter the blood [9]. Although feces were not collected in this study, the serum metabolome itself reflects microbial–host co-metabolism, as the blood metabolite profiles of germ-free and conventional mice differ markedly and the circulating levels of many indole derivatives, hippurate, and phenyl derivatives depend on the gut microbiota [49]. Notably, a series of studies on the same strain provides indirect support for this link. LP36 was isolated from the traditional Chinese fermented food “Suanshui,” and whole-genome sequencing together with in vitro evaluation confirmed its robust gastrointestinal tolerance, its colonization and adhesion capacity, and its antioxidant activity [24]. In a model of acute liver injury, LP36 restored gut microbiota balance, increasing the relative abundance of beneficial genera such as *Ligilactobacillus* and *Akkermansia* and reducing harmful genera such as *Alistipes* and *Parasutterella*, while its cecal metabolome was enriched in indole biosynthesis, bile secretion, and tyrosine metabolism [25]. In a model of alcoholic liver disease, LP36 likewise modulated gut microbiota composition and improved intestinal barrier function [19]. More importantly, metabolites derived from the intestinal contents of LP36-treated mice significantly downregulated the expression of inflammatory genes such as IL-1β and TNF-α [50], and a fecal microbiota transplantation experiment further showed that the protective effect of LP36 can be transferred through its reshaped microbiota [25]. This gut-level evidence from the same strain corresponds to the indole and bile-acid metabolites detected in the present study, consistent with a link between the gut and serum compartments within a single strain.

Among the differential metabolites, we identified several that are known to be produced or regulated by the gut microbiota. In the indole branch of tryptophan metabolism, the relative signal intensity of indole-3-carboxylic acid was higher after LPS and lower after LP36; such metabolites are produced mainly by bacterial tryptophan metabolism and can act as aryl hydrocarbon receptor ligands on astrocytes to limit central nervous system inflammation [51,52]. The kynurenine branch was likewise disturbed, with the relative signal intensity of L-kynurenine being higher after LPS and lower after high-dose LP36; this branch is jointly regulated by inflammation and the gut microbiota, and its neuroactive products can accumulate in the brain and are associated with depression [53,54]. Microbial degradation products of aromatic amino acids and polyphenols, such as *p*-hydroxyphenylacetylglycine and 4-vinylphenol sulfate, also showed LP36-associated shifts in relative signal intensity toward NS + NS group values; these are characteristic products of the bacterial degradation of aromatic amino acids, and the related 4-ethylphenyl sulfate has been confirmed to be of bacterial origin and to induce anxiety-like behavior on its own [55,56]. In addition, secondary bile acids that can be generated only by bacteria, such as taurodeoxycholate and ketolithocholic acid, were altered [57]; microbiota-related pathways including butanoate metabolism and the serotonergic synapse recurred in the LP36 groups, although these appeared only as uncorrected, trend-level results, and short-chain fatty acids are important microbiota-derived signals regulating the maturation and function of microglia [58].

These microbiota-related metabolites and the most prominent lipid remodeling observed in this study are unlikely to be independent of each other; both probably originate from the modulation of the gut microbiota by LP36. Secondary bile acids are simultaneously characteristic bacterial products and steroid/lipid molecules that participate in lipid absorption, transport, and metabolism, and they therefore serve as a natural hub linking microbial activity to host lipid remodeling. In addition, the gut microbiota can systemically influence host lipid homeostasis through short-chain fatty acids, bile-acid signaling, and the regulation of polyunsaturated fatty acid metabolism [38]. A parsimonious interpretation is therefore that LP36, by reshaping the gut microbiota, directly altered specific microbial metabolites such as indoles, kynurenines, and secondary bile acids. In parallel, it indirectly drove the lipid remodeling centered on polyunsaturated fatty acids and sphingolipids that constitutes the principal effect of this study. Because lipid metabolites are not exclusive to the gut microbiota and are also synthesized in large amounts by the host, the inference of microbial involvement is based mainly on these specific microbial metabolites, and the lipid remodeling is interpreted as a downstream, integrative consequence subject to microbial regulation, suggesting that reshaping the metabolic output of the gut microbiota may be an important mechanism by which LP36 alleviates depression-like behavior. However, the gut microbiota was not directly assessed in this study. Its involvement therefore remains a plausible hypothesis inferred from the serum metabolic signatures and the previously reported properties of this strain, rather than a mechanism demonstrated here. Direct verification will require further gut-microbiome and mechanistic studies in future work.

Several limitations should be acknowledged. First, the involvement of the microbiota–gut–brain axis was inferred from serum metabolic signatures rather than directly demonstrated; the gut microbiota, intestinal permeability, microbiota-derived metabolites, and peripheral or central inflammatory markers were not measured in this study. Second, metabolic profiling was performed on serum rather than feces—serum captures integrated cross-organ metabolism and matched the behavioral cohort, but fecal metabolomics would more directly reflect the functional output of the gut microbiota, and its absence limits direct mechanistic inference. Third, the LPS-induced model chiefly recapitulates depression with a strong inflammatory component and does not represent all subtypes of major depressive disorder, so extrapolation to patients—particularly non-inflammatory subtypes—should be made with caution. Fourth, only male mice were studied, precluding assessment of sex differences, and the experimental duration was relatively short. Fifth, the serum metabolome cannot be equated with metabolic changes in the brain, and no brain tissue was analyzed. Sixth, the per-group sample size for metabolomics was small (*n* = 3); although pooled-QC reproducibility was high (Pearson r > 0.99), this limits the statistical robustness and generalizability of the identified signatures, and these signatures should therefore be interpreted as hypothesis-generating rather than confirmatory. Seventh, metabolite annotations correspond to MSI level 2 or below rather than standard-confirmed identifications [30]. Eighth, the metabolites were extracted by a single methanol-based protein-precipitation step (80% methanol, diluted to a final methanol concentration of 53% before injection; Section 2.6). No single extraction protocol recovers all metabolite classes with equal efficiency, extraction recovery was not determined for any class in this study, and relative signal intensities are therefore not comparable between metabolite classes; the class composition of the profile reported here is shaped in part by the extraction chemistry. Ninth, pooled QC samples were injected at the beginning, middle, and end of the analytical sequence, so that no more than twelve study samples were analyzed between consecutive QC injections in this single 24-sample run; although the resulting QC reproducibility was high (Pearson r > 0.99), the denser QC insertion now recommended for longer analytical runs (e.g., every fifth injection) would provide finer-grained monitoring of instrument drift and will be adopted in future work. Finally, metabolomics provides correlative evidence, and whether the identified metabolites causally mediate the behavioral benefit of LP36 requires targeted validation. Future studies will integrate 16S rRNA sequencing, fecal and short-chain-fatty-acid profiling, targeted lipidomics, inflammatory-marker and brain-tissue measurements, and both sexes and larger cohorts, together with germ-free or fecal microbiota transplantation models, to clarify the causal mechanism by which LP36 alleviates LPS-induced depression-like behavior.

## 5. Conclusions

*Lacticaseibacillus paracasei* LP36 alleviated LPS-induced depression-like behavior in mice and broadly reshaped the serum metabolome. Its metabolic effect was largely a reverse regulation of the LPS-induced perturbation, concentrated in lipid-related pathways: the relative signal intensities of polyunsaturated fatty acids and sphingolipids were lower after LPS and higher after LP36 intervention. In parallel, the relative signal intensities of microbiota-derived metabolites, including indoles, kynurenines, and secondary bile acids, shifted toward control-group values after LP36 intervention. LP36 may therefore act, at least in part, through the microbiota–gut–brain axis.

## Data Availability

The raw mass spectrometry metabolomics data generated in this study have been deposited in the MetaboLights repository (https://www.ebi.ac.uk/metabolights/) (accessed on 23 July 2026) under accession number MTBLS14655 (https://www.ebi.ac.uk/metabolights/MTBLS14655) (accessed on 23 July 2026). All other data supporting the findings of this study are available within the article and its Supplementary Materials.

## Supplementary Materials

The following supporting information can be downloaded at: https://www.mdpi.com/article/10.3390/nu18152468/s1, Table S1: Behavioral paired *t*-tests (LP36-alone safety and model construction); Table S2: Day 28 two-way ANOVA and Tukey–Kramer post hoc tests; Table S3: Full serum metabolite quantification matrix (2867 metabolites × 24 samples); Table S4: Differential metabolites between NS + LPS and NS + NS; Table S5: KEGG enrichment for NS + LPS vs. NS + NS; Table S6: Differential metabolites between LPS + LP36 10^10^ and NS + LPS; Table S7: Differential metabolites split by chemical class; Table S8: KEGG enrichment of LP36 intervention and intersection pathways; Table S9: Direction annotation of the 180 shared differential metabolites. Figure S1: Dose-consistency of the LP36 serum metabolic effect. (A,B) PLS-DA score plots for the 1 × 10^8^ and 1 × 10^9^ LP36 doses versus the NS + LPS model group. (C) Concordance of the log2 fold-changes at the 1 × 10^8^ and 1 × 10^9^ doses with those at 1 × 10^10^, computed across the 601 metabolites called differential at 1 × 10^10^ (Spearman r = 0.95 and 0.92; 99% and 96% of metabolites changing in the same direction). (D) Key lipid mediators across the three doses. The numbers of differential metabolites versus NS + LPS were 494, 533, and 601 for the 1 × 10^8^, 1 × 10^9^, and 1 × 10^10^ doses, respectively.

## References

[1] GBD 2019 Mental Disorders Collaborators (2022). Global, regional, and national burden of 12 mental disorders in 204 countries and territories, 1990–2019: A systematic analysis for the Global Burden of Disease Study 2019. Lancet Psychiatry.

[2] Santomauro D.F., Mantilla Herrera A.M., Shadid J., Zheng P., Ashbaugh C., Pigott D.M., Cristiana A., Adolph C., Amlag J.O., Aravkin A. (2021). Global prevalence and burden of depressive and anxiety disorders in 204 countries and territories in 2020 due to the COVID-19 pandemic. Lancet.

[3] Beurel E., Toups M., Nemeroff C.B. (2020). The Bidirectional Relationship of Depression and Inflammation: Double Trouble. Neuron.

[4] Cipriani A., Furukawa T.A., Salanti G., Chaimani A., Atkinson L.Z., Ogawa Y., Leucht S., Ruhe H.G., Turner E.H., Higgins J.P.T. (2018). Comparative efficacy and acceptability of 21 antidepressant drugs for the acute treatment of adults with major depressive disorder: A systematic review and network meta-analysis. Lancet.

[5] Miller A.H., Raison C.L. (2016). The role of inflammation in depression: From evolutionary imperative to modern treatment target. Nat. Rev. Immunol..

[6] Dowlati Y., Herrmann N., Swardfager W., Liu H., Sham L., Reim E.K., Lanctôt K.L. (2010). A Meta-Analysis of Cytokines in Major Depression. Biol. Psychiatry.

[7] Dantzer R., O’Connor J.C., Freund G.G., Johnson R.W., Kelley K.W. (2008). From inflammation to sickness and depression: When the immune system subjugates the brain. Nat. Rev. Neurosci..

[8] Mo X., Guo S., He D., Cheng Q., Yang Y., Wang H., Ren Y., Liu L., Xie P. (2025). *Lactobacillus reuteri* DSM 17938 ameliorates LPS-induced depression-like and anxiety-like behaviors by modulating gut microbiota and brain metabolic function. Gut Pathog..

[9] Cryan J.F., O’Riordan K.J., Cowan C.S.M., Sandhu K.V., Bastiaanssen T.F.S., Boehme M., Codagnone M.G., Cussotto S., Fulling C., Golubeva A.V. (2019). The Microbiota-Gut-Brain Axis. Physiol. Rev..

[10] Foster J.A., McVey Neufeld K.-A. (2013). Gut–brain axis: How the microbiome influences anxiety and depression. Trends Neurosci..

[11] Radford-Smith D.E., Anthony D.C. (2023). Prebiotic and Probiotic Modulation of the Microbiota–Gut–Brain Axis in Depression. Nutrients.

[12] Dalile B., Van Oudenhove L., Vervliet B., Verbeke K. (2019). The role of short-chain fatty acids in microbiota–gut–brain communication. Nat. Rev. Gastroenterol. Hepatol..

[13] Dinan T.G., Stanton C., Cryan J.F. (2013). Psychobiotics: A Novel Class of Psychotropic. Biol. Psychiatry.

[14] Huang R., Wang K., Hu J. (2016). Effect of Probiotics on Depression: A Systematic Review and Meta-Analysis of Randomized Controlled Trials. Nutrients.

[15] Nikolova V., Cleare A., Young A., Stone J. (2021). Updated Review and Meta-Analysis of Probiotics for the Treatment of Clinical Depression: Adjunctive vs. Stand-Alone Treatment. J. Clin. Med..

[16] He B.-L., Cui R., Hu T.-G., Wu H. (2025). *Lactiplantibacillus plantarum* WH021 Alleviates Lipopolysaccharide-Induced Neuroinflammation via Regulating the Intestinal Microenvironment and Protecting the Barrier Integrity. J. Agric. Food Chem..

[17] Li W., Xu M., Liu Y., Zhang S., Wang J., Zhang Z., Xiao G., Wang R., Zhang J., Xue H. (2025). *Lactiplantibacillus plantarum* GOLDGUT-HNU082 Alleviates CUMS-Induced Depressive-like Behaviors in Mice by Modulating the Gut Microbiota and Neurotransmitter Levels. Foods.

[18] Tsai W.-H., Yeh W.-L., Chou C.-H., Wu C.-L., Lai C.-H., Yeh Y.-T., Liao C.-A., Wu C.-C. (2023). Suppressive Effects of *Lactobacillus* on Depression through Regulating the Gut Microbiota and Metabolites in C57BL/6J Mice Induced by Ampicillin. Biomedicines.

[19] Wang C., Chen X., Wang F., Chen T., Yin M., Liu Z., Li W., Zhu J. (2025). *Lacticaseibacillus paracasei* 36 Mitigates Alcoholic-Associated Liver Disease Through Modulation of Microbiota and AMPK Signaling. Nutrients.

[20] Wei C.-L., Wang S., Yen J.-T., Cheng Y.-F., Liao C.-L., Hsu C.-C., Wu C.-C., Tsai Y.-C. (2019). Antidepressant-like activities of live and heat-killed *Lactobacillus paracasei* PS23 in chronic corticosterone-treated mice and possible mechanisms. Brain Res..

[21] Xu M., Tian P., Zhu H., Zou R., Zhao J., Zhang H., Wang G., Chen W. (2022). *Lactobacillus paracasei* CCFM1229 and *Lactobacillus rhamnosus* CCFM1228 Alleviated Depression- and Anxiety-Related Symptoms of Chronic Stress-Induced Depression in Mice by Regulating Xanthine Oxidase Activity in the Brain. Nutrients.

[22] Zhang Y., Liang H., Wang Y., Cheng R., Pu F., Yang Y., Li J., Wu S., Shen X., He F. (2022). Heat-inactivated *Lacticaseibacillus paracasei* N1115 alleviates the damage due to brain function caused by long-term antibiotic cocktail exposure in mice. BMC Neurosci..

[23] McFarland L.V., Evans C.T., Goldstein E.J.C. (2018). Strain-Specificity and Disease-Specificity of Probiotic Efficacy: A Systematic Review and Meta-Analysis. Front. Med..

[24] Wang F., Li X., Wang Q., Jin Q., Fu A., Zhang Q., Yang R., Deng B., Li W. (2024). Based on whole genome sequencing and metabolomics analysis the safety and probiotic characteristics of *Lacticaseibacillus paracasei* 36 isolated from Chinese fermented vegetables. Food Biosci..

[25] Wang F., Wu L., Li X., Yao Y., Fu A., Yang H., Xiao Y., Li W. (2026). *Lacticaseibacillus paracasei* 36 attenuates D-GalN/LPS-induced acute liver injury in mice via suppressing the TLR4/NF-κB/MAPK pathway and NLRP3 inflammasome activation through modulating the intestinal microbiota. J. Adv. Res..

[26] Ahmad Azam A., Ismail I.S., Shaikh M.F., Abas F., Shaari K. (2021). Multi-Platform Metabolomics Analyses Revealed the Complexity of Serum Metabolites in LPS-Induced Neuroinflammed Rats Treated with Clinacanthus nutans Aqueous Extract. Front. Pharmacol..

[27] Willner P., Towell A., Sampson D., Sophokleous S., Muscat R. (1987). Reduction of sucrose preference by chronic unpredictable mild stress, and its restoration by a tricyclic antidepressant. Psychopharmacology.

[28] Porsolt R.D., Bertin A., Jalfre M. (1977). Behavioral despair in mice: A primary screening test for antidepressants. Arch. Int. Pharmacodyn. Ther..

[29] Steru L., Chermat R., Thierry B., Simon P. (1985). The tail suspension test: A new method for screening antidepressants in mice. Psychopharmacology.

[30] Sumner L.W., Amberg A., Barrett D., Beale M.H., Beger R., Daykin C.A., Fan T.W.-M., Fiehn O., Goodacre R., Griffin J.L. (2007). Proposed minimum reporting standards for chemical analysis: Chemical Analysis Working Group (CAWG) Metabolomics Standards Initiative (MSI). Metabolomics.

[31] Westerhuis J.A., Hoefsloot H.C.J., Smit S., Vis D.J., Smilde A.K., van Velzen E.J.J., van Duijnhoven J.P.M., van Dorsten F.A. (2008). Assessment of PLSDA cross validation. Metabolomics.

[32] Bharti V., Bhardwaj A., Hood K., Elias D.A., Metcalfe A.W.S., Kim J.S. (2021). A systematic review and meta-analysis of lipid metabolomic signatures of Major Depressive Disorder. J. Psychiatr. Res..

[33] Bazinet R.P., Layé S. (2014). Polyunsaturated fatty acids and their metabolites in brain function and disease. Nat. Rev. Neurosci..

[34] Liao Y., Xie B., Zhang H., He Q., Guo L., Subramanieapillai M., Fan B., Lu C., McIntyre R.S. (2019). Efficacy of omega-3 PUFAs in depression: A meta-analysis. Transl. Psychiatry.

[35] Giacobbe J., Benoiton B., Zunszain P., Pariante C.M., Borsini A. (2020). The Anti-Inflammatory Role of Omega-3 Polyunsaturated Fatty Acids Metabolites in Pre-Clinical Models of Psychiatric, Neurodegenerative, and Neurological Disorders. Front. Psychiatry.

[36] Serhan C.N. (2014). Pro-resolving lipid mediators are leads for resolution physiology. Nature.

[37] Wang Y., Dong L., Pan D., Xu D., Lu Y., Yin S., Wang S., Xia H., Liao W., Sun G. (2022). Effect of High Ratio of n-6/n-3 PUFAs on Depression: A Meta-Analysis of Prospective Studies. Front. Nutr..

[38] Schoeler M., Caesar R. (2019). Dietary lipids, gut microbiota and lipid metabolism. Rev. Endocr. Metab. Disord..

[39] Levy B.D., Clish C.B., Schmidt B., Gronert K., Serhan C.N. (2001). Lipid mediator class switching during acute inflammation: Signals in resolution. Nat. Immunol..

[40] Regulska M., Szuster-Głuszczak M., Trojan E., Leśkiewicz M., Basta-Kaim A. (2020). The Emerging Role of the Double-Edged Impact of Arachidonic Acid-Derived Eicosanoids in the Neuroinflammatory Background of Depression. Curr. Neuropharmacol..

[41] Deyama S., Kaneda K., Minami M. (2025). Resolution of depression: Antidepressant actions of resolvins. Neurosci. Res..

[42] Brunkhorst-Kanaan N., Klatt-Schreiner K., Hackel J., Schröter K., Trautmann S., Hahnefeld L., Wicker S., Reif A., Thomas D., Geisslinger G. (2019). Targeted lipidomics reveal derangement of ceramides in major depression and bipolar disorder. Metabolism.

[43] Dinoff A., Herrmann N., Lanctôt K.L. (2017). Ceramides and depression: A systematic review. J. Affect. Disord..

[44] Brown H.A., Thomas P.G., Lindsley C.W. (2017). Targeting phospholipase D in cancer, infection and neurodegenerative disorders. Nat. Rev. Drug Discov..

[45] Gui S.-W., Liu Y.-Y., Zhong X.-G., Liu X.-Y., Zheng P., Pu J.-C., Zhou J., Chen J.-J., Zhao L.-B., Liu L.-X. (2018). Plasma disturbance of phospholipid metabolism in major depressive disorder by integration of proteomics and metabolomics. Neuropsychiatr. Dis. Treat..

[46] Ke H., Bai F., Li Z., Zhu Y., Zhang C., Li Y., Talifu Z., Pan Y., Liu W., Xu X. (2024). Inhibition of phospholipase D promotes neurological function recovery and reduces neuroinflammation after spinal cord injury in mice. Front. Cell. Neurosci..

[47] Kaufmann F.N., Costa A.P., Ghisleni G., Diaz A.P., Rodrigues A.L.S., Peluffo H., Kaster M.P. (2017). NLRP3 inflammasome-driven pathways in depression: Clinical and preclinical findings. Brain Behav. Immun..

[48] Ren Y., Ren Z., Zhao S., Wu W., Wang J., He F., Zhong Q., Zhang H., Chen J., Xu K. (2025). Chronic administration of isotretinoin induces depressive- and anxiety-like behaviors by altering the neuroactive ligand-receptor interaction pathway in adolescent mice. Transl. Psychiatry.

[49] Wikoff W.R., Anfora A.T., Liu J., Schultz P.G., Lesley S.A., Peters E.C., Siuzdak G. (2009). Metabolomics analysis reveals large effects of gut microflora on mammalian blood metabolites. Proc. Natl. Acad. Sci. USA.

[50] Wang F., Li X., Wang Q., Jin Q., Xu S., Tang L., Zeng Z., Fu A., Zhu J., Zhang Q. (2025). Screening of lactic acid bacteria strains from Chinese fermented food (Suanshui) and its protective effect on acute liver injury in mice. Probiotics Antimicrob. Proteins.

[51] Roager H.M., Licht T.R. (2018). Microbial tryptophan catabolites in health and disease. Nat. Commun..

[52] Rothhammer V., Mascanfroni I.D., Bunse L., Takenaka M.C., Kenison J.E., Mayo L., Chao C.-C., Patel B., Yan R., Blain M. (2016). Type I interferons and microbial metabolites of tryptophan modulate astrocyte activity and central nervous system inflammation via the aryl hydrocarbon receptor. Nat. Med..

[53] Cervenka I., Agudelo L.Z., Ruas J.L. (2017). Kynurenines: Tryptophan’s metabolites in exercise, inflammation, and mental health. Science.

[54] O’Mahony S.M., Clarke G., Borre Y.E., Dinan T.G., Cryan J.F. (2015). Serotonin, tryptophan metabolism and the brain-gut-microbiome axis. Behav. Brain Res..

[55] Dodd D., Spitzer M.H., Van Treuren W., Merrill B.D., Hryckowian A.J., Higginbottom S.K., Le A., Cowan T.M., Nolan G.P., Fischbach M.A. (2017). A gut bacterial pathway metabolizes aromatic amino acids into nine circulating metabolites. Nature.

[56] Hsiao E.Y., McBride S.W., Hsien S., Sharon G., Hyde E.R., McCue T., Codelli J.A., Chow J., Reisman S.E., Petrosino J.F. (2013). Microbiota modulate behavioral and physiological abnormalities associated with neurodevelopmental disorders. Cell.

[57] Sayin S.I., Wahlström A., Felin J., Jäntti S., Marschall H.-U., Bamberg K., Angelin B., Hyötyläinen T., Orešič M., Bäckhed F. (2013). Gut microbiota regulates bile acid metabolism by reducing the levels of tauro-beta-muricholic acid, a naturally occurring FXR antagonist. Cell Metab..

[58] Erny D., Hrabě de Angelis A.L., Jaitin D., Wieghofer P., Staszewski O., David E., Keren-Shaul H., Mahlakõiv T., Jakobshagen K., Buch T. (2015). Host microbiota constantly control maturation and function of microglia in the CNS. Nat. Neurosci..

