# Supplementary material for: Lacticaseibacillus paracasei LP36 Alleviates Lipopolysaccharide-Induced Depression-like Behavior in Mice: A Serum Metabolomics Study
Source: Nutrients. 2026 Jul 29;18(15):2468. doi: 10.3390/nu18152468 (PMC13467607; doi:10.3390/nu18152468)
Supplement: Supplementary file 1 [file nutrients-18-02468-s001.zip › Figure_S1.pdf]

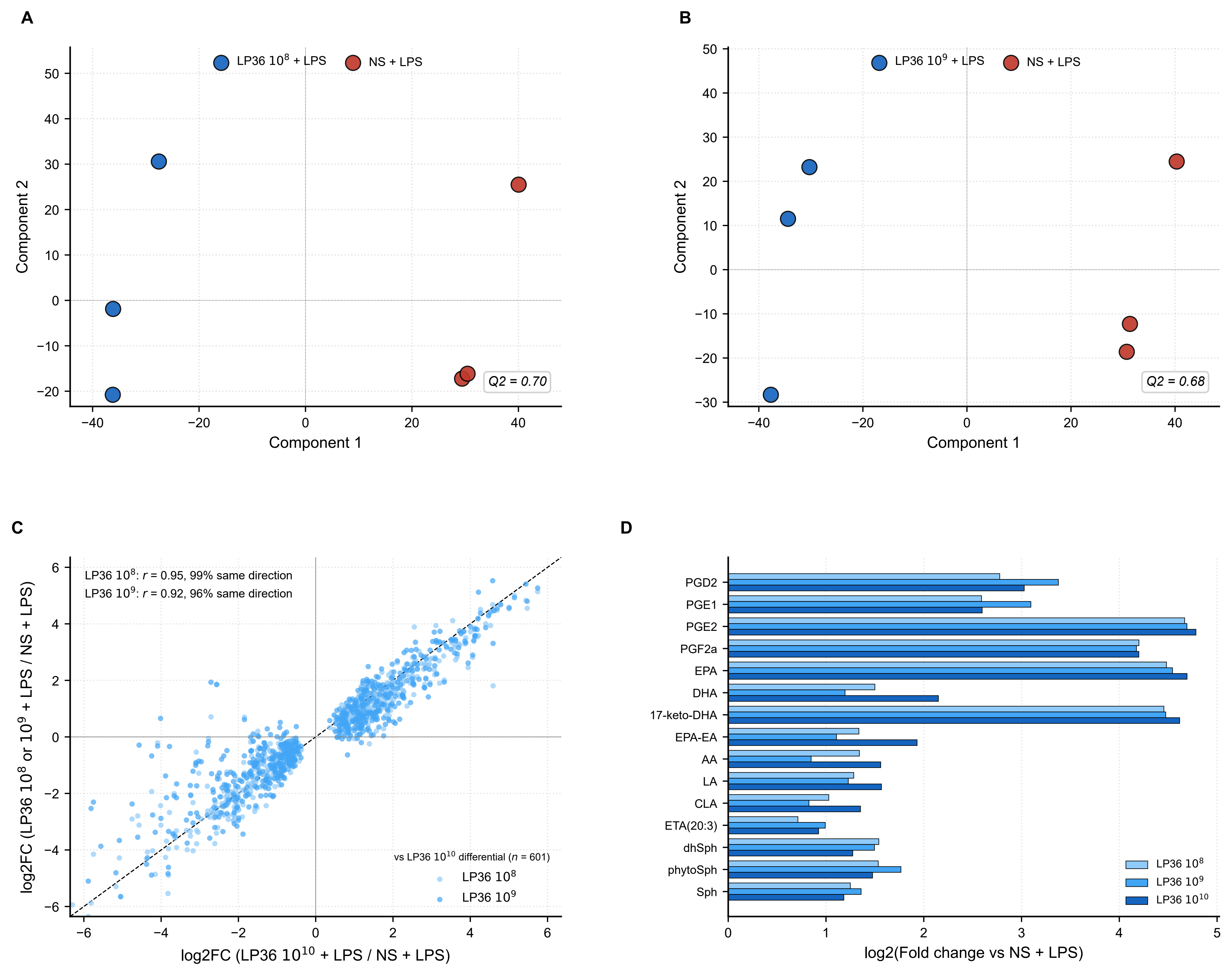
A
LP36 10^8 + LPS
NS + LPS
Q2 = 0.70
Component 1
Component 2
B
LP36 10^9 + LPS
NS + LPS
Q2 = 0.68
Component 1
Component 2
C
LP36 10^8: r = 0.95, 99% same direction
LP36 10^9: r = 0.92, 96% same direction
vs LP36 10^{10} differential (n = 601)
LP36 10^8
LP36 10^9
log2FC (LP36 10^{10} + LPS / NS + LPS)
log2FC (LP36 10^8 or 10^9 + LPS / NS + LPS)
D
PGD2
PGE1
PGE2
PGF2a
EPA
DHA
17-keto-DHA
EPA-EA
AA
LA
CLA
ETA(20:3)
dhSph
phytoSph
Sph
LP36 10^8
LP36 10^9
LP36 10^{10}
log2(Fold change vs NS + LPS)
